# Tools and Approaches for Studying Microglia *In vivo*

**DOI:** 10.3389/fimmu.2020.583647

**Published:** 2020-10-07

**Authors:** Elisa Eme-Scolan, Samantha J. Dando

**Affiliations:** ^1^École Normale Supérieure de Lyon, Université Claude Bernard Lyon I, Université de Lyon, Lyon, France; ^2^Faculty of Health, Centre for Immunology and Infection Control, School of Biomedical Sciences, Queensland University of Technology (QUT), Brisbane, QLD, Australia

**Keywords:** microglia, central nervous system, brain, retina, reporter mice, microglia homeostatic genes, microglia imaging, microglia depletion

## Abstract

Microglia are specialized resident macrophages of the central nervous system (CNS) that have important functions during neurodevelopment, homeostasis and disease. This mini-review provides an overview of the current tools and approaches for studying microglia *in vivo*. We focus on tools for labeling microglia, highlighting the advantages and limitations of microglia markers/antibodies and reporter mice. We also discuss techniques for imaging microglia *in situ*, including *in vivo* live imaging of brain and retinal microglia. Finally, we review microglia depletion approaches and their use to investigate microglial function in CNS homeostasis and disease.

## Introduction

The CNS (comprising the brain parenchyma, spinal cord, and neural retina) is populated with specialized resident macrophages called microglia. Microglia are derived from yolk sac progenitors (1) and are long lived cells that are maintained within the CNS through *in situ* self-renewal (2). Microglia continuously survey their surroundings via highly motile processes (3) and are exquisitely programmed to respond to changes in their microenvironment. In response to injury, infection or inflammation, microglia become “activated” and can shift into numerous functional states to elicit innate immune responses. In addition to performing immune functions, microglia are intimately involved in neurodevelopment and maintaining homeostasis of the healthy CNS. Some of their “non-immune” functions include: phagocytosing apoptotic/dead neural cells and debris (4); supporting neurogenesis, neuronal development and neural circuit assembly (5–7); inducing synapse formation (8); maintaining synaptic structure and function (9); synaptic pruning (10, 11); and maintaining neurons via the formation of somatic junctions (12).

The heterogeneous states of activated microglia exist on a continuum ranging from neuroprotective to neurotoxic/pathogenic (13). There is increasing evidence, largely from animal studies, that uncontrolled activated microglia contribute to the pathogenesis of a range of neurological and ocular diseases, including Alzheimer's disease (AD) (14), multiple sclerosis (15), Parkinson's disease (16), Huntington's disease (17), Amyotrophic Lateral Sclerosis (ALS) (18), neuromyelitis optica (19) and autoimmune uveitis (20). However, protective disease-associated microglia have also been described in AD and ALS (21), and may also exist in retinal degeneration (22). Despite the ongoing debate regarding the protective vs. pathogenic role of microglia, they are clearly involved in a wide range of CNS diseases and display a high level of plasticity.

Microglia are the subject of intense research efforts; however, there are several challenges associated with studying these cells. *Challenge 1*: microglia cultured *in vitro* do not recapitulate *in vivo* microglia in their physiological environment. Although important advances have been made to develop new microglia culture methods, including serum-free culture conditions and iPSC-derived microglia [reviewed in (23–25)], *in vitro* approaches that reflect microglia within their immune-privileged neural environment are still lacking. *Challenge 2*: when studying microglia *in vivo*, manipulation of the CNS (for example, preparing brain slices) can lead to injury and subsequent microglia activation (26), which is a limitation for studying microglia in their physiological state. *Challenge 3*: microglia share overlapping markers with other myeloid cells (27). It is essential to differentiate microglia from border-associated macrophages (BAMs), which reside within the meninges, choroid plexus and perivascular spaces of the brain, and the choroid that lies adjacent to the retina. Similarly, microglia must also be distinguished from circulating myeloid cells that infiltrate the CNS during neuroinflammation.

**Figure 1:**
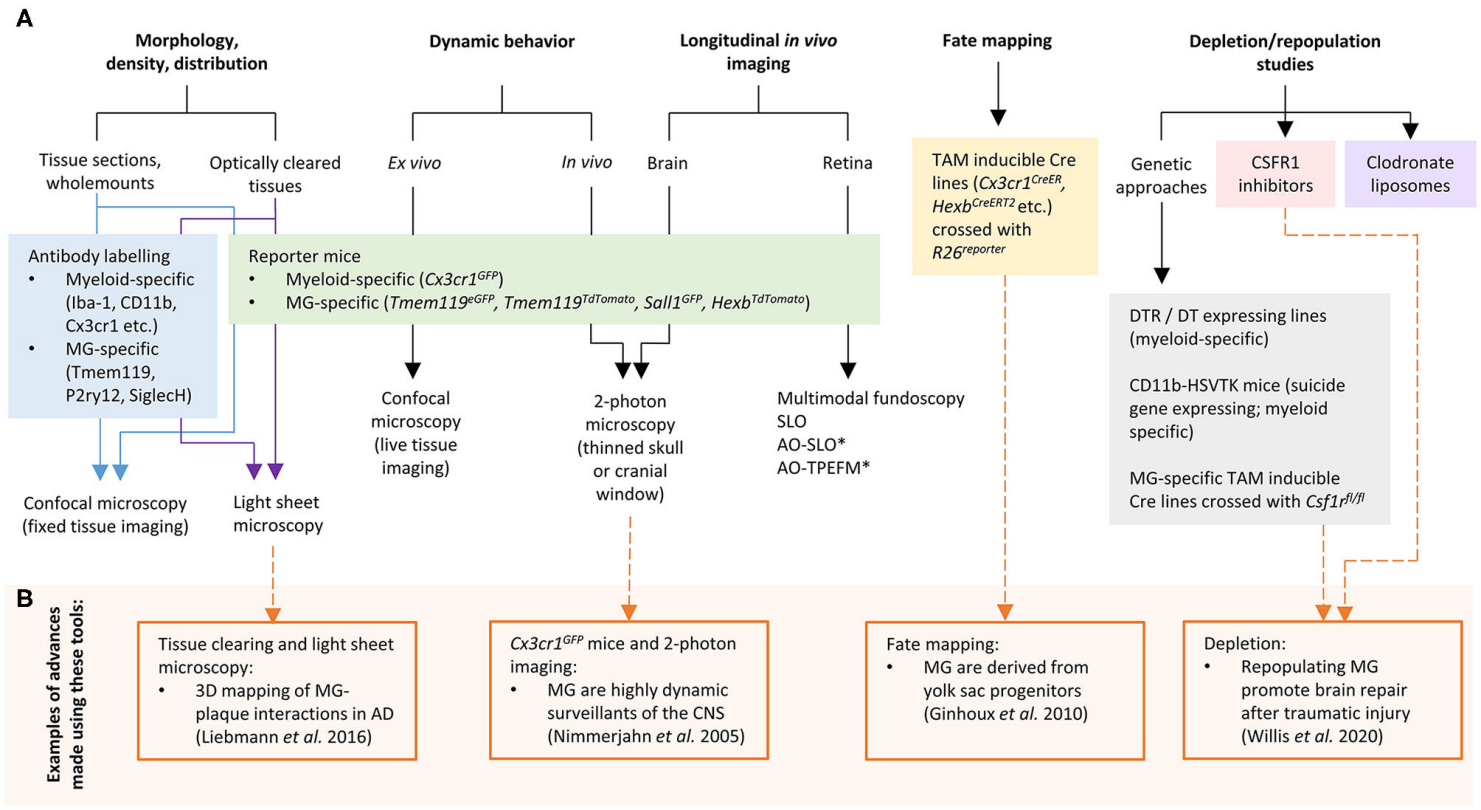
**(A)** Tools and approaches for studying microglia *in vivo*. **(B)** Examples of advances in microglia research that have been made using these tools. AD, Alzheimer's disease; AO, adaptive optics; CSF1R, colony stimulating factor 1 receptor; DT, diphtheria toxin; DTR, diphtheria toxin receptor; MG, microglia; SLO, scanning laser ophthalmoscopy; TPEFM, two-photon excitation fluorescence microscopy. *can also be used for *in vivo* retinal microglia dynamic behavior studies.

In recent years, significant progress has been made to address these challenges by developing new cellular and molecular tools for microglia research. In this mini-review we discuss the current “microglia tool kit” for *in vivo* research (summarized in Figure 1 and Table 1), and how recently developed approaches can be used to overcome some of the above challenges.

**Table 1:** Advantages, limitations, and applications of tools to study microglia *in vivo*.

	**Advantages**	**Limitations**	**Applications for studying MG *in vivo***
**LABELING MG**			
**Myeloid markers** (Iba-1, Cx3cr1, CD45, CD11b)	Antibodies widely available; most work well in fixed tissue sections and whole mounts.	Also expressed by BAMs and peripheral immune cells.	Phenotyping (e.g., analysis of MG density, distribution, morphology, marker co-expression, cell interactions).
**MG-specific markers** (Tmem119, P2RY12, FCRLS, SiglecH, HexB)	Expression restricted to MG in healthy brain. Highly expressed by MG in steady state.	Expression may be decreased by MG during disease. Tmem119 expression may not be restricted to MG in the diseased retina. Few commercially available antibodies.	
***Cx3cr1*** **reporter mice***Cx3cr1^*GFP*^; Cx3cr1^*CreER*^:R26 ^*Reporter*^*	GFP or Cre under control of *Cx3cr1* promoter, which is highly expressed in homeostatic MG. *Cx3cr1^*GFP*^* mice available on C57Bl/6 and BALB/c background. *Cx3cr1^*CreER*^:R26 ^*Reporter*^* mice: Temporary labeling of peripheral myeloid cells; irreversible labeling of MG.	*Cx3cr1^*GFP*^*: BAMs and peripheral immune cells also labeled. Heterozygous *Cx3cr1^*GFP*^* mice may have partially impaired Cx3cl1-Cx3cr1 signaling compared to WT mice. *Cx3cr1^*CreER*^:R26 ^*Reporter*^*: BAMs also labeled.	*Cx3cr1^*GFP*^*: Phenotyping. Homozygous *Cx3cr1^*GFP*^* mice can be used to study effects of full *Cx3cr1* deletion. *Cx3cr1^*CreER*^:R26 ^*Reporter*^*: Fate mapping MG in development, disease, and aging.
**“Next generation” MG reporter mice** *Tmem119^*Reporter*^, Sall1^*GFP*^, Hexb^*TdTomato*^* *Tmem119/Sall1/Hexb/P2ry12^*CreER*^: R26 ^*Reporter*^*	Higher specificity for MG compared to *Cx3cr1* lines. Hexb reporter stably expressed during neurodegeneration and demyelination.	Non-specific recombination can occur in some Cre lines, resulting in subsets of BAMs and glia also being labeled. Fluorescent reporter expression may be decreased during disease.	Phenotyping (fluorescent reporter lines); fate mapping in development, disease, and aging (Cre lines).
**IMAGING MG**			
**Confocal microscopy** (Fixed tissues)	High resolution 3D datasets generated by collecting optical Z sections through tissue. Many laboratories have access to confocal microscopes through core facilities.	Most confocal microscopes have limited imaging depth: requires specimen to be sectioned (brain) or microdissected (retina). Image acquisition can be slow. Photobleaching of tissue can occur. Cannot study dynamic behavior of MG in fixed tissues. Fixation may affect MG morphology.	Imaging fluorescently labeled microglia in fixed brain/spinal cord/retinal sections or whole mounts.
**Tissue clearing and light sheet microscopy** (Fixed tissues)	Can perform rapid 3D reconstructions of optically cleared tissues (deep imaging). Eliminates requirement for histological sectioning. Large variety of tissue clearing methods for mouse brain and eye; some compatible with antibody labeling and endogenous fluorescent reporters.	Not all research facilities have access to light sheet microscopes and specialized objectives. Some hydrophobic tissue clearing methods quench fluorescent reporter signals.	Imaging fluorescently labeled microglia in fixed, optically cleared tissues (“global” tissue imaging).
***In vivo*** **live imaging** Two-photon microscopy (brain) Multimodal fundoscopy, SLO, AO-SLO (retina)	Imaging MG in live animals.	Specialized instrumentation required; not available in all research facilities. High level of technical expertise required. Microglia process dynamics are increased in anesthetized mice compared to awake mice. Cranial window or thinned skull preparation required for brain two-photon imaging. Not all brain regions are accessible using two-photon imaging.	Imaging dynamic MG behavior *in vivo* (study MG tissue surveillance functions). *In vivo* longitudinal imaging of MG. *In vivo* cellular interactions.
**DEPLETING MG**			
**Clodronate liposomes**	Effective for short-term depletion studies.	MG depletion requires intracerebral or intravitreal injection (break “immune privilege” due to physical trauma). Likely to also deplete BAMs. Off-target effects.	Depletion of MG to determine their functions in development, homeostasis or disease. Study MG repopulation.
**CSF1R inhibitors** (PLX3397, PLX5662)	Cross the blood-brain/blood-retina barrier and can be administered orally. 90–99% MG depletion after 21 days treatment. Can be used for sustained MG depletion studies. MG repopulate quickly after cessation of treatment.	Not MG-specific	
**Genetic approaches** *Cx3cr1^*CreER*^:R26^*iDTR*^ Iba1-tTA::DTA^*tetO*/*tetO*^ CD11b-HSVTK Siglech^*DTR*/*DTR*^ Sall1^*CreER*^Csf1r^*fl*/*fl*^ Hexb^*CreERT*2/*CreERT*2^Csf1r^*fl*/*fl*^*	*Siglech^*DTR*/*DTR*^, Sall1*^*CreER*^*Csf1r*^*fl*/*fl*^ and *Hexb*^*CreERT*2/*CreERT*2^*Csf1r*^*fl*/*fl*^ mice enable precise depletion of MG (BAMs or circulating leukocytes not targeted). High levels of MG depletion (>90%) can be achieved in *Cx3cr1^*CreER*^:R26^*iDTR*^, Iba1-tTA::DTA^*tetO*/*tetO*^* and *CD11b-HSVTK* mice.	Depletion requires injections of either tamoxifen, DT or ganciclovir. *Cx3cr1^*CreER*^:R26^*iDTR*^, Iba1-tTA::DTA^*tetO*/*tetO*^* and *CD11b-HSVTK* mice do not enable MG-specific depletion (BAMs and subsets of circulating myeloid cells also depleted) Depletion less efficient in *Hexb^*CreERT*2/*CreERT*2^Csf1r^*fl*/*fl*^* mice.	

*AO, adaptive optics; BAMs, border-associated macrophages; CSF1R, colony stimulating factor 1 receptor; DT, diphtheria toxin; MG, microglia; SLO, scanning laser ophthalmoscopy*.

## Tools for Labeling Microglia in the CNS

### Markers and Antibodies

Immunohistochemistry and flow cytometry are common techniques in neuroimmunology. Traditionally, Iba-1 antibodies have been used to label/stain microglia using immunohistochemistry; however, Iba-1 is also expressed by BAMs and subsets of peripheral myeloid cells (28, 29). During neuroinflammation, peripheral Iba-1^+^ myeloid cells that invade the CNS adopt a similar morphology to activated microglia (30), therefore microglia cannot be definitively distinguished from infiltrating leukocytes based solely on Iba-1 expression. Microglia also express several other markers that are common to BAMs and peripheral myeloid cells, including CD45, CD11b, CD68, Cx3cr1, F4/80, and CSF1R (31). Using flow cytometry, it is possible to distinguish microglia from other leukocytes based on their unique expression profile of selected surface markers. For example, microglia in the healthy CNS express low/intermediate levels of CD45, whereas BAMs and peripheral immune cells are CD45^hi^. Whether microglia retain low/intermediate CD45 expression during neuroinflammation is debated and appears to be disease- and CNS region-dependent. O'Koren et al. reported that retinal microglia retain a CD45^lo^ phenotype in a light injury model and can be distinguished from infiltrating myeloid cells based on their unique CD45^lo^ CD11c^lo^ F4/80^lo^ I-A/I-E^−^ signature (32). In contrast, Plemel et al. demonstrated that spinal cord microglia increased CD45 expression in a demyelination model and suggested that classical markers (such as CD45 and Cx3cr1) are less sensitive for distinguishing activated microglia from infiltrating myeloid cells in this model (33). Therefore, more recent studies have focused on identifying microglia-specific markers that can reliably distinguish microglia from other leukocytes during health and disease.

Bulk RNA-seq studies have identified several highly expressed genes that constitute the “microglia homeostatic signature” (28, 34–36). These signature genes, including *Tmem119, P2ry12, Olfml3, Hexb, Fcrls, Siglech, Tgfbr1, Gpr34, Sall1*, and others, have been the basis for the development of a range of new tools for microglia research in recent years, including microglia-specific antibodies. Tmem119 antibodies were developed by Bennett et al. for specific labeling of microglia using immunohistochemistry and flow cytometry (37). Tmem119 is expressed by mouse and human microglia but not BAMs or peripheral immune cells, and commercially available Tmem119 antibodies are now widely used. Butovsky et al. generated antibodies to P2ry12 and FCRLS, and showed that these distinguished mouse microglia from infiltrating myeloid cells (34). Furthermore, Konishi et al. reported that SiglecH antibodies specifically labeled microglia in the mouse brain but not BAMs or infiltrating monocytes (38). Microglia signature genes have also recently been used as targets for single molecule fluorescence *in situ* hybridization (smFISH) using RNAscope assays. Hammond et al. used smFISH (alongside single cell RNA-seq) to study microglia throughout the mouse lifespan and demonstrated that (i) microglia universally expressed FCRLS and (ii) diverse subtypes of microglia with unique spatial gene expression signatures exist in the developing, aged, and injured mouse brain (39).

The development of microglia-specific antibodies is useful for labeling these cells during homeostatic conditions. However, these markers may not be reliable for the identification of microglia during development and disease. For example, embryonic microglia do not express Tmem119, and expression of this protein by all microglia does not occur until postnatal day 14 in mice (37). This limits the use of Tmem119 antibodies during development and early postnatal stages. Furthermore, Tmem119 immunoreactivity may not be restricted to microglia in the diseased retina, as Su et al. reported that other cell types (such as Müller cells) may become Tmem119^+^ in a choroidal neovascularization model (40). Although further studies are required to validate these findings in other experimental models, Tmem119 antibodies should be used cautiously in the diseased retina. Moreover, microglial homeostatic genes including *Tmem119, P2ry12*, and *Siglech* are reported to be downregulated in CNS diseases (21, 41, 42), which may further limit their use. This is likely to be disease-dependent however, as some studies have shown stable expression of microglia homeostatic genes during disease (43). Given the range of markers and antibodies that can be used to identify microglia, the choice of targets needs to be carefully considered for each research question.

### Reporter Mice

The fractalkine receptor gene *Cx3cr1* is the basis of several reporter mouse lines that are widely used in microglia research. In *Cx3cr1*^*GFP*^ mice, microglia express GFP under the control of the *Cx3cr1* promoter (44). Homeostatic microglia express high levels of Cx3cr1 and are therefore strongly GFP^+^, which is extremely useful for identifying microglia within tissues. One limitation of these mice is that they are either heterozygous (*Cx3cr1*^*GFP*/+^) or deficient (*Cx3cr1*^*GFP*/*GFP*^) for *Cx3cr1*, and therefore neuron-microglia communication (mediated by Cx3cl1-Cx3cr1 signaling) is impaired compared to wild type mice, which can lead to reduced cognitive and synaptic function (45). Another important caveat is that Cx3cr1 is also expressed by BAMs, peripheral monocytes, DCs and NK cells (44), therefore GFP expression is not restricted to microglia. In contrast, tamoxifen inducible *Cx3cr1*^*CreER*^ mice crossed with *R26*^*Reporter*^ mice are a useful tool to distinguish microglia and CNS-infiltrating myeloid cells (32, 46). In these mice, CreER is constitutively expressed under the control of the *Cx3cr1* promoter, whereas *R26*^*Reporter*^ expression is dependent on Cre recombination. Upon tamoxifen administration, Cre is activated in Cx3cr1 expressing cells and a stop codon controlling *R26*^*Reporter*^ expression is excised, resulting in Cx3cr1^+^ cells becoming R26^Reporter+^ (i.e., both tissue resident macrophages and circulating myeloid cells express *R26*^*Reporter*^). Due to the temporary activation of Cre and the turnover of circulating myeloid cells, several weeks after tamoxifen induction blood-borne myeloid cells lose *R26*^*Reporter*^expression. In contrast, *R26*^*Reporter*^ labeling is irreversible in long-lived tissue resident macrophages, including microglia (32, 46). However, BAMs are also long-lived resident cells that retain expression of the tamoxifen-induced reporter using this *Cx3cr1*^*CreER*^ approach (47), and additional means are required to distinguish microglia from BAMs.

Recently described reporter mice have taken advantage of microglia-specific signature genes. *Tmem119*^*eGFP*^ (48), *Tmem119*^*TdTomato*^ (49), *Sall1*^*GFP*^ (50, 51), and *Hexb*^*TdTomato*^ (52) mice are knock-in strains in which expression of fluorescent reporter proteins is largely restricted to microglia. Although *Tmem119, Sall1*, and other microglia signature genes are typically downregulated during CNS disease, mouse microglia were recently reported to maintain stable *Hexb* expression in models of neurodegeneration and demyelination, suggesting that *Hexb*^*TdTomato*^ mice may be suitable for consistent bright labeling of microglia in both homeostasis and CNS disease (52). In addition to the *Cx3cr1*^*CreER*^ mice described above, several other tamoxifen inducible Cre lines now also exist for microglia fate mapping and genetic manipulation. These include *Tmem119*^*CreERT*2^ (48), *Sall1*^*CreER*^ (50), *Hexb*^*CreERT*2^ (52) and *P2ry12*^*CreER*^ (53). Although these mouse strains have a much higher specificity for microglia compared to *Cx3cr1*^*CreER*^ mice, they still have some limitations. For example, *Tmem119*^*CreERT*2^ and *P2ry12*^*CreER*^ mice are not 100% microglia specific as recombination also occurs in some BAM subsets after tamoxifen administration. In addition, recombination is detected in glial cells in *Sall1*^*CreER*^ mice (52, 54). Of the available tamoxifen inducible Cre lines, *Hexb*^*CreERT*2^ mice are reported to have the highest microglia specificity, although not all microglia are targeted (brain region dependent) and recombination also occurs in a very small percentage of perivascular macrophages (52). Despite these limitations, these “next generation” reporter mice are an excellent resource for the neuroimmunology research community.

## Techniques for Imaging Microglia *In Situ*

### Confocal Microscopy of Fixed and Fresh Tissue

The ability to label microglia using the above approaches enables researchers to visualize these cells *in situ*. Confocal laser scanning microscopy is frequently used to image fluorescently labeled microglia in tissue sections (fixed), retinal wholemounts (fixed or fresh) and organotypic brain slices (fresh) to investigate microglial density, morphology, distribution, cellular interactions and dynamic behavior. However, there are limitations to these approaches. Fixation obviously preserves the tissue but makes it impossible to study the dynamism of microglia. Fixation procedures can also have effects on microglia morphology (55), which may confound some experiments. On the other hand, the dynamic behavior of microglia can be observed in fresh retinal wholemounts (56) and brain slices (57) but as tissues are placed in artificial medium for live imaging, microglia are likely to sense perturbations within their environment. In addition, the process of preparing brain slices and retinal wholemounts for live imaging can cause physical trauma. Therefore, microglia observed using live *ex vivo* approaches may not recapitulate their *in vivo* physiological counterparts.

### Tissue Clearing and Light Sheet Microscopy

Tissue clearing techniques coupled with light sheet microscopy can be used to visualize microglia within intact transparent CNS tissues. Tissue clearing methods [reviewed in detail in (58)] make tissues transparent, resulting in minimal light scattering and enabling deep imaging and 3D reconstruction of tissues (including brains and eyes). This allows unbiased global investigation of tissues and eliminates the requirement to perform histological sectioning, which is a major advantage of this technique. A range of tissue clearing methods (including hydrophobic, hydrophilic and hydrogel-based methods) can be used to “clear” fixed CNS tissues (58, 59). Depending on the clearing method, tissues can be labeled with antibodies, nanobodies and lectins prior to clearing for visualization of cells and structures of interest. For example, Liebmann et al. used iDISCO clearing and Iba-1 immunolabeling to reconstruct the 3D interactions between brain microglia, vasculature and amyloid-β plaques in a mouse model of Alzheimer's disease (60). Some hydrophobic tissue clearing methods [such as 3DISCO (61)] rapidly quench fluorescent reporter protein signals; however, several hydrophilic and hydrogel-based clearing approaches effectively preserve endogenous fluorescence and can be used to image microglia in fluorescent reporter mice (described above). For instance, Xu et al. developed the FACT tissue clearing method and applied this to *Cx3cr1*^*YFP*^ mouse brains for deep imaging of YFP^+^ microglia (62). Using this method, the authors detected fluorescent signal to a Z depth of ~800 μm although the signal intensity was markedly diminished after ~550 μm (62).

Unlike the brain, the eye is naturally transparent. However, the retinal pigment epithelium, the interface between the retinal photoreceptors and the choroid, is highly melanized (darkly pigmented), which remains a challenge for standard clearing methods. Specialized clearing techniques including EyeCi (63), DEEP-Clear (64), and EyeDISCO (65) were developed to address this issue and enable light sheet imaging of retinas within intact eyeballs. However, to the best of our knowledge these techniques have not yet been used for retinal microglia studies.

### *In vivo* Live Imaging of Microglia

Two-photon microscopy is a powerful technique that allows unparalleled imaging of microglia in live animals. Time-lapse *in vivo* two-photon imaging of *Cx3cr1*^*GFP*^ mouse brains led to the seminal discovery that microglia are highly dynamic surveillants that constantly extend and retract their processes during homeostasis (3). Several studies have since used two-photon imaging to study microglial dynamism in CNS disease models and aging (66–68). Most two-photon microglia imaging studies are performed on mice under general anesthesia through either a cranial window or thinned skull preparation [reviewed in detail in (69)]. The major advantage of this approach is that it is performed in live animals and therefore the cells are observed within their physiological environment. However, there are some important limitations. Firstly, microglial arbor, surveillance territory and process dynamics are increased in anesthetized mice compared to awake mice (70, 71) and methods to image microglia in awake mice may be required for some studies. Secondly, cranial window surgery and skull thinning procedures can induce CNS damage and subsequent microglial activation (69). Thirdly, not all brain regions are accessible using this approach. It remains difficult to image deep brain regions, therefore most two-photon studies are performed on cortical microglia. Using *Hexb*^*TdTomato*^ mice, Masuda et al. demonstrated that it is possible to perform time lapse *in vivo* two-photon imaging of microglia to a depth of 500 μm from the surface of the brain (52).

Specialized tools are available for *in vivo* live imaging of retinal microglia. Multimodal fundoscopy and scanning laser ophthalmoscopy (SLO) enable longitudinal studies of microglia in fluorescent reporter mice (72, 73) but these methods do not have sufficient resolution for examination of microglia fine process morphology or dynamic behavior, nor do they provide any depth information. Adaptive optics (AO) is a recent advancement that corrects optical aberrations and significantly improves resolution. When combined with SLO, AO-SLO enables microglia within distinct retinal layers to be resolved *in vivo* (74). Miller et al. demonstrated that AO-SLO can be used to quantify the 3D distribution, morphology and dynamism of microglia within individual layers of the *Cx3cr1*^*GFP*^ mouse retina (75). More recently, Qin et al. developed an adaptive optics two-photon excitation fluorescence microscopy (AO-TPEFM) system that provides further improvements in the resolution of *in vivo* retinal microglia imaging (76). Combined with microglia-specific fluorescent reporter mice, AO-SLO and AO-TPEFM imaging will be useful tools to examine *in vivo* longitudinal changes in microglia in retinal diseases.

*In vivo* microglia imaging in humans remains challenging due to a lack of specific tools but positron emission tomography (PET) combined with 18-kDa translocator protein (TPSO)-binding radioligands is used to non-specifically assess microglial activation and neuroinflammation in patients *in vivo* (77, 78). The use of this clinical neuroimaging technique in multiple sclerosis patients has been reviewed previously (77) and will not be discussed here.

## Tools for Depleting Microglia

### Clodronate Liposomes

To attribute *in vivo* functions to microglia, depletion studies are often required. One approach to deplete microglia *in vivo* involves clodronate liposomes. Clodronate liposomes are phagocytosed by macrophages and subsequently released into the cytosol, where clodronate inhibits mitochondrial ADP/ATP translocase and induces macrophage apoptosis (79). To deplete microglia, clodronate liposomes are injected intracerebrally (80, 81) or intravitreally (82) to bypass the blood-brain/blood-retina barrier. This approach is effective for short-term microglia depletion; however, intracerebral injections can induce physical trauma and break “immune privilege” of the CNS (83). Moreover, off-target effects may occur (84), and control liposomes (that do not contain clodronate) have been shown to non-specifically activate microglia (85). Intracerebral delivery of clodronate liposomes is also likely to deplete BAMs (in particular, perivascular macrophages), although the specificity of depletion following intracerebral administration of clodronate liposomes has not been investigated to the best of our knowledge. In contrast, intraventricular injection of clodronate liposomes results in selective depletion of BAMs and not microglia (86).

### CSF1R Inhibitors

CSF1R inhibitors are effective for microglia depletion, as adult microglia depend on CSF1R for survival (87). Unlike CSF1R antibodies and clodronate liposomes, CSF1R inhibitors are small molecules that readily cross the blood-brain/blood-retina barrier and can be administered orally, either by oral gavage or mixed within standard rodent chow. PLX3397 and PLX5662 are the most commonly used CSF1R inhibitors for microglia depletion as these have high potency (PLX3397) (87) and high brain penetrance (PLX5662) (88). CSF1R inhibitors are effective to achieve long-term, sustained microglial depletion *in vivo*. However, this approach does not eliminate 100% of microglia. For example, PLX3397 depletes 90–99% of microglia from the mouse brain after 21 days of treatment (89). Microglial repopulation begins after CSF1R inhibitor treatment ceases, and it is thought that repopulating microglia are generated from the few microglial cells that are not eliminated by the treatment (72). In the mouse retina, repopulated microglia display similar dynamism to endogenous microglia, suggesting that repopulated microglia restore homeostatic tissue surveillance (72). Interestingly, the adult mouse brain only has capacity for a single microglia repopulation event, as following repeated PLX3397 depletion cycles microglia fail to repopulate the brain (90). Using PLX3397 depletion, Spangenberg et al. reported that microglial elimination improved contextual memory deficits in a mouse model (5xFAD) of Alzheimer's disease (91). Further studies demonstrated that sustained PLX5662 microglia depletion impaired plaque formation in 5xFAD mice (88, 92). As such, CSF1R inhibitors are being investigated as a potential treatment option for CNS diseases (93). However, studies involving CSF1R depletion strategies must be interpreted cautiously, as CSF1R inhibitors also likely target BAMs and some peripheral immune populations (93).

### Genetic Approaches

Microglia depletion can also be achieved with genetic approaches. In *Cx3cr1*^*CreER*^*:R26*^*iDTR*^ mice, microglia and other long-lived resident immune cells (but not circulating myeloid cells) express the diphtheria toxin receptor (DTR) following tamoxifen administration. Upon exposure to diphtheria toxin (DT), ~99% of microglia are depleted within 1 day, and low numbers of microglia are maintained in the CNS for up to 7 days post-DT (46). *Iba1-tTA::DTA*^*tetO*/*tetO*^ mice also take advantage of a DT system. In these mice, selective expression of DT occurs in Iba-1^+^ cells after withdrawal of doxycycline. This results in ~90% depletion of microglia (94); however, Iba-1^+^ BAMs and peripheral myeloid cells are also likely to be targeted in these mice. Depletion systems based on the *CD11b* promoter also broadly target myeloid cells and are not microglia specific. In *CD11b-HSVTK* mice, expression of the suicide gene *HSVTK* is driven by the *CD11b* promoter. After administration of the drug ganciclovir, HSVTK converts ganciclovir to a toxic compound, which induces apoptosis of CD11b^+^ cells. Intracerebroventricular infusion of ganciclovir achieves high levels (<90%) of microglia depletion; however, this is short-lived as microglia robustly repopulate the CNS within 2 weeks of ganciclovir treatment (95).

Genetic depletion systems targeting microglia-specific genes offer a more precise approach to microglia elimination without affecting BAMs and circulating myeloid cells. Using *Siglech*^*DTR*/*DTR*^ mice, Konishi et al. reported that ~80–85% of Iba-1^+^ CD206^−^ microglia were depleted in the cortex and area postrema following intraperitoneal DT injection. In contrast, the numbers of perivascular macrophages, meningeal macrophages and infiltrating monocytes following nerve injury were unaffected (38). Tamoxifen inducible Cre lines also exist for specific microglia depletion, including *Sall1*^*CreER*^*Csf1r*^*fl*/*fl*^ and *Hexb*^*CreERT*2/*CreERT*2^*Csf1r*^*fl*/*fl*^ mice. In these strains, the conditional *Csf1r* allele is deleted by tamoxifen inducible Cre expressed from the *Sall1* or *Hexb* locus (50, 52). Whilst these strains specifically target microglia (and not BAMs or circulating myeloid cells), recombination appears to be less efficient that other genetic depletion systems. For example, in *Hexb*^*CreERT*2/*CreERT*2^*Csf1r*^*fl*/*fl*^ mice only 60% of microglia are depleted after tamoxifen injection (52). Moreover, in *Sall1*^*CreER*^*Csf1r*^*fl*/*fl*^ mice small numbers of Sall1^+^ CD45^−^ cells in the adult kidney and liver are also depleted (50). Whilst tamoxifen-induced depletion of these cells in adult mice does not result in overt inflammation in the serum, spleen, liver, and kidneys (50), it is possible there may be other effects in these peripheral organs.

## Conclusion

Researchers have previously applied traditional immunological approaches to study microglia the CNS. Thanks to rapid advances in the neuroimmunology field, we now have a suite of tools and techniques for microglia research. The discovery of microglia-specific signature genes has elevated the choice of markers, antibodies and reporter mice for studying these cells *in vivo*. However, further research to identify microglia-specific markers that are stably expressed during neuroinflammation is required. The combination of microglia-specific labeling, powerful *in vivo* imaging, and precise depletion approaches is already accelerating discoveries relating to microglial biology. These tools provide opportunities to investigate microglial function and phenotype *in vivo*, and to understand the spatial and disease-dependent heterogeneity of these multitasking, multifunctional cells.

## Author Contributions

EE-S and SD conceived and researched data for the article and wrote the review. All authors contributed to the article and approved the submitted version.

## Conflict of Interest

The authors declare that the research was conducted in the absence of any commercial or financial relationships that could be construed as a potential conflict of interest.
